# Modeling nervous system tumors with human stem cells and organoids

**DOI:** 10.1186/s13619-022-00150-7

**Published:** 2023-03-01

**Authors:** Jie Duan, Yuan Wang

**Affiliations:** Department of Neurosurgery, State Key Laboratory of Biotherapy and Cancer Center, West China Hospital, Sichuan University and National Collaborative Innovation Center, Chengdu, 610041 China

**Keywords:** Nervous system tumors, Human stem cells, Organoids, Tumor models, Tumor cell-of-origin, Tumor organoids, 3D multicellular coculture, Genome editing

## Abstract

Nervous system cancers are the 10th leading cause of death worldwide, many of which are difficult to diagnose and exhibit varying degrees of treatment resistance. The limitations of existing cancer models, such as patient-derived xenograft (PDX) models and genetically engineered mouse (GEM) models, call for the development of novel preclinical cancer models to more faithfully mimic the patient’s cancer and offer additional insights. Recent advances in human stem cell biology, organoid, and genome-editing techniques allow us to model nervous system tumors in three types of next-generation tumor models: cell-of-origin models, tumor organoids, and 3D multicellular coculture models. In this review, we introduced and compared different human stem cell/organoid-derived models, and comprehensively summarized and discussed the recently developed models for various primary tumors in the central and peripheral nervous systems, including glioblastoma (GBM), H3K27M-mutant Diffuse Midline Glioma (DMG) and H3G34R-mutant High-grade Glioma (HGG), Low-grade Glioma (LGG), Neurofibromatosis Type 1 (NF1), Neurofibromatosis Type 2 (NF2), Medulloblastoma (MB), Atypical Teratoid/rhabdoid Tumor (AT/RT), and meningioma. We further compared these models with PDX and GEM models, and discussed the opportunities and challenges of precision nervous cancer modeling with human stem cells and organoids.

## Background

Primary nervous system tumors, whether noncancerous (benign) or cancerous (malignant), arise from the nervous tissues in the brain, spinal cord, and peripheral nerves (Louis et al. 2021). Nervous system cancers are the 10th leading cause of death for men and women (Siegel et al. 2022). Many of these cancers are difficult to diagnose and exhibit varying degrees of treatment resistance, partly due to the unique anatomic locations where they originate and the often infiltrative nature of cancer cells. Thus, there is an urgent need to develop novel diagnostics and therapies based on a deeper understanding of these cancers.

Preclinical models are invaluable tools for studying various aspects of tumor development. Other than established cancer cell lines, the most commonly used preclinical models in the field are patient-derived xenograft (PDX) models and genetically engineered mouse (GEM) models (Aparicio et al. 2015; Day et al. 2015). PDX models are established by implanting patients’ tumor cells into immunodeficient or humanized mice. These models closely mimic human cancer, can be easily passaged and expanded, and are suitable for drug screening. However, due to patient-specific genetic background and alterations, even PDX models for the same cancer type may differ dramatically from patient to patient (Aparicio et al. 2015). Moreover, since they are derived from full-blown cancers, they cannot model the de novo tumorigenesis from the cell(s) of origin for cancer. GEM models, on the other hand, introduce defined genetic modifications into specific cell types to drive tumorigenesis, and have been used as a gold standard to functionally validate the cancer drivers and the cell(s) of origin for cancer (Day et al. 2015). However, they are, after all, murine tumors that may exhibit cross-species differences and often do not reflect the complex heterogeneity of human cancer. In addition, it is time-consuming to establish GEM models through multi-generation crossing, and tumor development is often slow and variable, precluding their use for drug screening. While there are many PDX and GEM models for nervous system cancers, the limitations of existing cancer models call for the development of novel preclinical cancer models to address these problems.

Recent advances in human stem cell biology, organoid, and genome-editing techniques allow us to directly model tumorigenesis from human stem/progenitor cells by introducing defined initiating cancer drivers (Hockemeyer and Jaenisch 2016; Papapetrou 2016; Qian et al. 2019). These techniques are particularly well-established for the nervous system. Patient-derived cancer organoid has also emerged as a personalized, scalable strategy to model cancer in a dish. In this review, we summarize various techniques to model nervous system tumors with human stem cells and/or organoids, highlight new findings with these models, and discuss the opportunities and challenges for precision cancer modeling for nervous system tumors.

## Different types of human stem cell/organoid-derived tumor models

**Fig.1:**
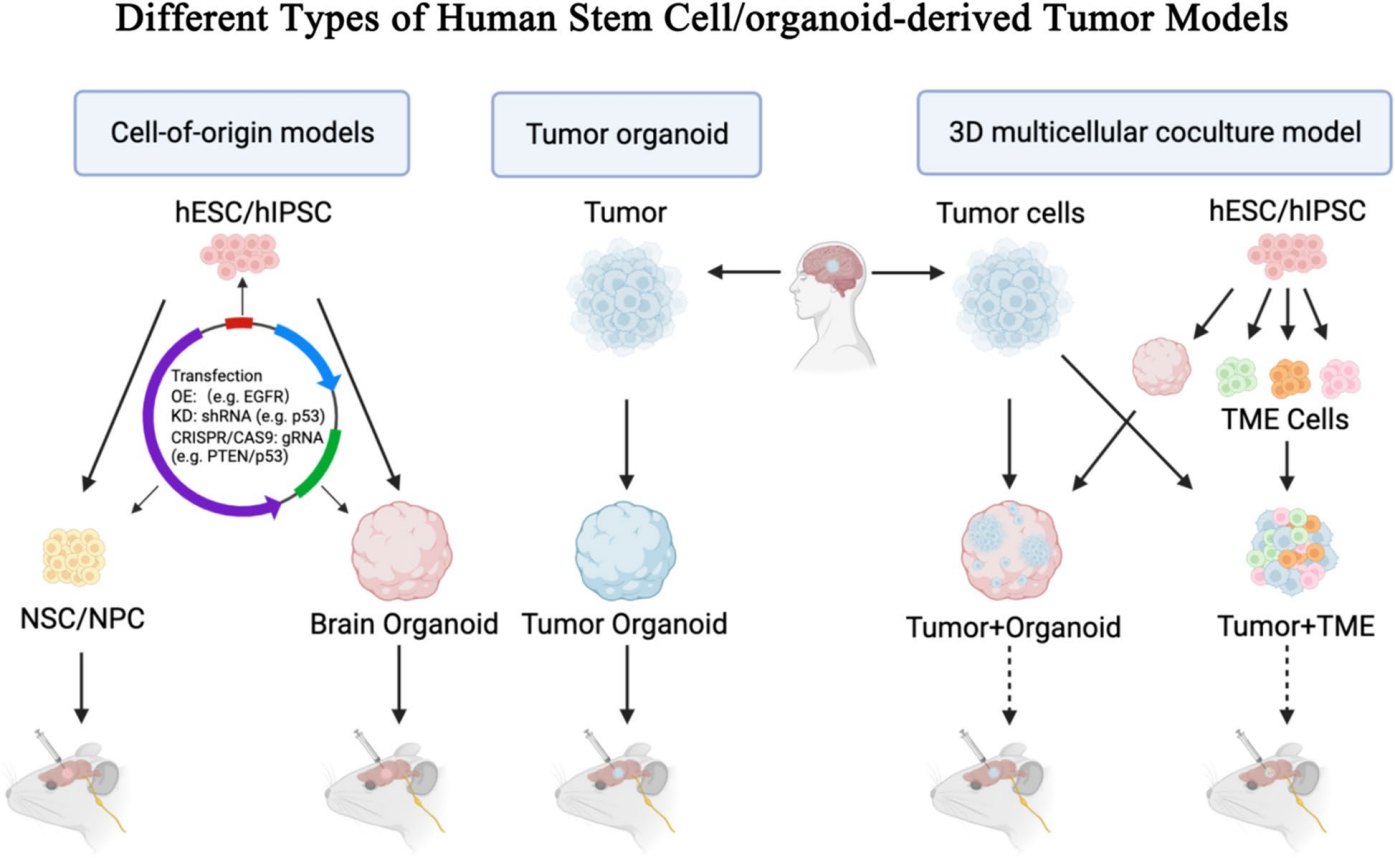
Different types of human stem cell/organoid-derived tumor models. Cell-of-origin models are established by introducing cancer drivers into hESCs/hIPSCs, NSCs/NPCs, or their derivative brain organoids, often through overexpression of oncogenes, CRISPR-mediated genome editing, and/or RNAi knockdown of tumor suppressors using viral vectors. Tumor organoid: Tumor organoids are directly generated from patient-derived tumor tissues or tumor cells. 3D multicellular coculture model: Patient-derived tumor cells are co-cultured with hESC/hIPSC-derived brain organoids or tumor microenvironmental (TME) cells to form 3D multicellular systems. All these systems can be subsequently transplanted into immunocompromised mice to form tumors in vivo.

The new generation of stem cell or organoid-derived tumor models can be generally grouped into three categories: cell-of-origin models, tumor organoids, and 3D multicellular coculture models (Fig. 1). Each of these models serves different purposes for cancer research and has unique advantages over existing preclinical tumor models.

### Cell-of-origin models

Cell-of-origin models are established by engineering cancer drivers into normal stem/progenitor cells or brain organoids through viral or non-viral transduction, which serve as the cell(s)-of-origin for cancer (Visvader 2011). For certain germline mutation-driven familiar cancer types, mutant stem/progenitor cells and organoids can be obtained from patient-derived induced pluripotent stem cells (IPSCs) (Papapetrou 2016). These genetically modified/germline-mutant stem/progenitor cells or brain organoids can be subsequently transplanted into immunocompromised/humanized mice to generate tumors in vivo. In many ways, these models are similar to GEM models. Like GEM models, they can be used to determine which cell type is most susceptible to malignant transformation under a defined genetic context. Moreover, these models have major advantages over GEM models: 1) They directly model tumorigenesis from human cells, 2) They can efficiently introduce and test the tumorigenicity of multiple potential cancer drivers/regulators in different cell types in a relatively short period of time, without the need of multi-generation crossing for GEM models. One main disadvantage of these models is that they often lack an intact immune environment since they require xenograft transplantation to generate tumors in vivo (Papapetrou 2016). This is especially true in brain tumor modeling. One major constituent of the brain tumor immune microenvironment is microglia, which are deficient even in human hematopoietic stem/progenitor cell-reconstituted, humanized mice (Keane et al. 2021). Recently developed human microglia transplant mouse models and microglia-containing brain organoids may help address this challenge (Fattorelli et al. 2021; Hasselmann et al. 2019; Mancuso et al. 2019), but is yet to be tested in brain tumor modeling.

### Tumor organoids

Unlike cell-of-origin models that start from genetically modified normal cells, tumor organoids are directly generated from human tumor tissues. Under three-dimensional (3D) culture conditions, neoplastic cells from patient samples can self-assemble into organoids that mimic key histopathological, genetic, and phenotypic characteristics, including the treatment response of the parental tumors (LeSavage et al. 2022). Tumor organoids can be viewed as a 3D intermediate between cancer cell lines and PDX models. The success rate of organoid generation is generally higher than traditional cancer cell lines or cancer stem cell lines. Moreover, they maintain subclonal tumor cell diversity, extracellular matrix, and at least a portion of tumor microenvironmental cells, which are missing in traditional 2D cell culture (LeSavage et al. 2022).

Recent studies have begun to rigorously test what clinical aspects tumor organoids can mimic the parental tumors at bulk and single-cell levels. LeBlanc et al. found that patient-derived GBM explants are genetically similar to parental tumors, retain their transcriptional characteristics, and exhibit similar levels of transcriptional heterogeneity (LeBlanc et al. 2022). In contrast, glioma stem cell (GSC) lines appear to select for a subpopulation of tumor cells with more homogeneous transcriptional profiles. Shakya et al. used spatial-capture RNA-sequencing to show that GBM organoids not only retain cells at distinct transcriptional states, but also mimic the transition between nutrient-rich (organoid rim) and nutrient-poor pseudopalisading/perinecrotic tumor zones (organoid core) (Shakya et al. 2021). These studies demonstrate that tumor organoids can faithfully mimic the genetics, transcriptional states, and possibly necrotic tumor niche of the parental tumors. However, tumor organoids alone may not accurately mimic more complex tumor niches, such as the vascular, immune, or neuronal niches, since many of these microenvironmental cells may be lost during the culture process (LeSavage et al. 2022).

Since tumor organoids are derived from established tumors, they cannot model de novo tumor development from cancer-initiating cells, but rather offers a fast and scalable way to characterize and test the behavior of tumors from individual patients. One main disadvantage of these models is a lack of normal brain tissue microenvironment. To address this problem, researchers have attempted to orthotopically inject tumor organoids into immunodeficient mice to rigorously test their behavior in a more physiologically relevant setting. For instance, orthotopically transplanted glioma organoids have been shown to integrate with the host brain and exhibit neovascularization with host vasculature (Golebiewska et al. 2020; Ogawa et al. 2018).

### 3D Multicellular coculture models

Human stem cell/organoid-based 3D multicellular coculture models are established by co-culturing cancer stem cells or cancer cell lines in 3D with normal tissue organoids such as brain organoids (Fiorini et al. 2020), or multiple human pluripotent stem cell-derived cell types that constitute the tumor microenvironment. In the tumor-organoid coculture settings, cancer cells often deeply invade and proliferate within the host organoids, resulting in mosaic “tumor in the organoid”. While these models have shown promise as a platform to study the tumor microenvironment, their usefulness largely depends on to what extent the host organoids mimic the in vivo tissue microenvironment. Take brain organoids for example, they usually lack mature oligodendrocytes, functional mature neurons, vascular and immune cells, all of which are essential components of the brain tumor microenvironment (Qian et al. 2019). To partly address this problem, researchers have taken a reductionist approach to coculture cancer cells with known microenvironmental cells differentiated from human stem cells (Plummer et al. 2019; Tang et al. 2020), which offers a compelling alternative strategy to model the tumor microenvironment.

## Human stem cell/organoid-derived models for nervous system tumors

The past few years have seen a rapid development of nervous system tumor models using human stem cells/organoids. We performed an exhaustive search in PubMed with keywords “stem cell”, “organoid”, along with nervous system tumor types based on the latest WHO classifications (Louis et al. 2021), while excluding traditional cancer stem cells studies, resulting in over 40 recently published literatures (Table 1). Here, we present a comprehensive review of different models for various types of nervous system tumors, along with the major findings and new insights they provide.

**Table 1:** Human stem cell/organoid-derived models for nervous system tumors

Tumor Types	Model Types	Model Subtypes	Model Descriptions	References
Glioblastoma (GBM)	Cell-of-origin models	Stem cell model	p53KD IPSC line–p53KD NPC p53KD NPC: Lentivirus expressing Ras/EGFR/Src	Sancho-Martinez et al. 2016
			ESC: Transcription activator-like effector nuclease (TALEN)-mediated homologous recombination (HR) to delete PTEN^Δ1^–NSC	Duan et al. 2015
			Hues8-iCas9 hPSCs– iCas9 hNSCs iCas9 NSC: CRISPR/Cas9-mediated TP53/NF1/PTEN knockout	Wang et al. 2021
			IPSC: CRISPR/Cas9-mediated PTEN and NF1 knockout IPSC: CRISPR/Cas9-mediated TP53 and PDGFRA^Δ8–9^ knockout	Koga et al. 2020
		Organoid model	hESC—organoid, CRISPR/ Cas9 target an HRasG12V-IRES-tdTomato into the TP53 locus Organoid-derived tumor cell or primary patient-derived glioblastoma cell co-culture with organoid	Ogawa et al. 2018
			Organoid: Sleeping Beauty (SB) transposon-mediated gene insertion for oncogene-amplification and CRISPR/Cas9-based mutagenesis of tumor suppressor genes. The cancer drivers used include MYC^OE^, CDKN2A^−^/CDKN2B^−^/EGFR^OE^/EGFRvIII^OE^, NF1^−^/PTEN^−^/TP53^−^, EGFRvIII^OE^/ CDKN2A^−^/PTEN^−^	Bian et al. 2018
	Tumor organoids		Patient-derived GBM cell lines–GBM organoid	Hubert et al. 2016; Jacob et al. 2020; Golebiewska et al. 2020; Shakya et al. 2021
			Patient-derived GBM cell lines–GBM explant	LeBlanc et al. 2022
	3D multicellular coculture model		IPSC/hESC–organoid Cancer cell co-cultured with organoid	Krieger et al. 2020; Choe et al. 2020
			IPSC/hESC–organoid Cancer stem cell co-cultured with organoid	Goranci-Buzhala et al. 2020; Linkous et al. 2019
			IPSC–organoid PTPRZ1-positive tumor cells co-cultured with organoid	Bhaduri et al. 2020
			IPSC–neurons/glial cells/ astrocyte Cancer cells co-cultured with neurons/glial cells/ astrocyte	Plummer et al. 2019
			Cancer stem cell co-cultured with 3D-bioprinted tumor microevironmental cells	Tang et al. 2020
Diffuse Midline Glioma, H3K27M-mutant (DMG) and H3G34R-mutant High-grade Glioma (HGG)	Cell-of-origin models	Stem cell model	IPSC—iNSC: CRISPR/cas9-mediated H3.3K27M point mutation + shTP53	Haag et al. 2021
			hESC–NPC NPC: Lentivirus expressing PDGFRA/H3.3K27M/shTP53	Funato et al. 2014
			Hindbrain NSC: piggyBac H3.3-K27M Forebrain NSC: piggyBac H3.3-G34R/PDGFRA and CRISPR/Cas9-mediated TP53 knockout	Bressan et al. 2021
			hESC: CRISPR/Cas9-mediated TP53/ATRX knockout and Lentivirus expressing H3.3-G34R mutation—ventral forebrain NPC or ventral hindbrain NPC	Funato et al. 2021
Low-grade Glioma (LGG)	Cell-of-origin models	Stem cell model	hESC–NSC NSC: Lentivirus expressing R132H-IDH and shP53/shATRX	Modrek et al. 2017
	Tumor organoids		Patient-derived lower-grade glioma cell lines–lower-grade glioma organoid	Abdullah et al. 2022
Neurofibromatosis Type 1 (NF1)	Cell-of-origin models	Stem cell model	IPSC: CRISPR/Cas9-mediated NF1 knockout–Neural crest–Schwann cell Patient-derived NF1-/- plexiform neurofibroma cells–NF1-/- IPSC-Neural crest–Schwann cell	Mazuelas et al. 2022
			IPSC: CRISPR/Cas9-mediated NF1 knockout–Schwannian lineage cells	Mo et al. 2021
Medulloblastoma	Cell-of-origin models	Stem cell model	IPSC–NES NES: transduction with MYCN Patients with Gorlin syndrome–IPSC–NES	Huang et al. 2019
			IPSC–NPC NPC: lentivirus expressing MYC and DNp53	Xue et al. 2021
			Embryonic hindbrain NES/IPSC–NSC NSC: lentivirus expressing MYCN	Čančer et al. 2019
			NSC: lentiviral and retroviral vectors expressing MYC/DNp53/hTERT/AKT	Hanaford et al. 2016
			Gorlin syndrome patient cell lines (SHH receptor PTCH1 mutation)–IPSC–iNES	Susanto et al. 2020
		Organoid model	IPSC–cerebellar organoid (piggyBac expressing c-MYC/Otx2)	Ballabio et al. 2020
	Tumor organoids		Patient-derived MB cells-MB organoid	Frisira et al. 2019; Li et al. 2022
Atypical Teratoid/rhabdoid Tumor (AT/RT)	Cell-of-origin models	Stem cell model	IPSC: CRISPR/Cas9-mediated SMARCB1/TP53 knockout–NPC	Terada et al. 2019
Meningiomas	Tumor organoids		Patient-derived meningioma cell–meningioma organoid	Yamazaki et al. 2021

### Glioblastoma (GBM)

GBM is the most common primary malignant brain tumor that often occurs in the frontal and temporal lobes of adult brains (Louis et al. 2021). GBM is highly infiltrative and resistant to standard chemo/radiation therapies, with a median survival of 15 months after treatment. GBM is among the best molecularly characterized cancer types (Sturm et al. 2014), and by far has the most human stem cell/organoid-derived models among all nervous system tumor types (Table 1).

#### Cell-of-origin Models for GBM

Bulk and single-cell genomics and transcriptomics have revealed the extreme inter- and intra-tumor heterogeneity in GBM (Bhaduri et al. 2020; Brennan et al. 2013; Neftel et al. 2019; Patel et al. 2014; Verhaak et al. 2010; Wang et al. 2019; Wang et al. 2017). Different transcriptional subgroups of GBM enrich for distinct combinations of cancer drivers, including frequent mutations in *TP53*, *PTEN, CDKN2A, NF1, EGFR, IDH1*, and *TERT* promoter*,* as well as *EGFR*, *PDGFRA, CDK4* amplification (Brennan et al. 2013). The most likely cell(s) of origin for GBM are neural stem/progenitor cells (NSCs/NPCs) in the subventricular zone (SVZ) and oligodendrocyte precursor cells (OPCs), as demonstrated by genetic studies on GEM models for GBM and human SVZ cells in patients with GBM (Alcantara Llaguno et al. 2009; Lee et al. 2018; Liu et al. 2011; Llaguno et al. 2019; Wang et al. 2009; Zhu et al. 2005). These findings lay the ground for using genetically modified human NSCs or brain organoids to establish cell-of-origin models for GBM.

Duan et al. and Sancho-Martinez et al. were among the first to establish cell-of-origin models for GBM using human embryonic stem cells (ESCs) or induced pluripotent stem cells (IPSCs) (Duan et al. 2015; Sancho-Martinez et al. 2016). Duan et al. used TALEN-mediated homologous recombination to delete the exon 1 of *PTEN* in human ESCs, while Sancho-Martinez et al. knocked down *TP53* and overexpressed Ras/EGFR/SRC in IPSCs through lentiviral infection. Genetically modified ESCs or IPSCs were subsequently differentiated into NSCs, transplanted into the striatum of immunocompromised mice, and developed tumors in vivo. More recently, Koga et al. and our group developed IPSC or human NSC-derived models that histologically and transcriptionally mimics human GBM, by introducing more clinically relevant mutational combinations (*TP53*/*PDGFRA*, *PTEN*/*NF1*, *TP53*/*PTEN/NF1, TP53/NF1, and TP53/PTEN*) (Koga et al. 2020; Wang et al. 2021*).* These human stem cell-derived GBM models are generally useful for mechanism studies and drug screening. When combined with single-cell transcriptomics, these models revealed the malignant transformation trajectory from mutant NSCs to GBM (Wang et al. 2021), and the longitudinal evolution of GBM between serial passages and transplantations (Koga et al. 2020).

In 2018 Bian et al. and Ogawa et al. established human organoid-based cell-of-origin models for GBM (Bian et al. 2018; Ogawa et al. 2018). Remarkably, Bian et al. tested the oncogeniety of 18 brain cancer driver combinations by monitoring targeted organoid cells labeled by a GFP reporter (Bian et al. 2018). Only four cancer driver combinations exhibited dramatic overgrowth. Among them, *MYC* overexpression (*MYC*^OE^) transcriptionally and cellularly resembled primitive neuroectodermal tumors of the central nervous system (CNS-PNETs), while the remaining three (*CDKN2A*^*–/–*^*/CDKN2B*^*–/–*^*/EGFR*^*OE*^*/EGFRvIII*^*OE*^, *TP53*^*–/–*^*/PTEN*^*–/–*^*/NF1*^*–/–*^, *EGFRvIII*^*OE*^*/CDKN2A*^*–/–*^*/PTEN*^*–/–*^) resembled GBM. Similarly, Ogawa et al. used *HRas*^G12V^ knockin into the *TP53* locus to generate highly invasive GBM-like tumor cells in the brain organoids (Ogawa et al. 2018). These organoid-derived tumor cells are suitable for preclinical drug screening, and can be isolated and transplanted into immunocompromised mice to generate tumors in vivo.

#### Tumor organoids for GBM

In 2016 Hubert et al. reported the first 3D culture system that supports long-term growth and expansion of tumor organoids derived directly from dissociated/minced glioblastoma specimens (Hubert et al. 2016). Jacob et al. further improved the protocol to rapidly (within 1–2 weeks) generate GBM organoids (named GBOs) directly from fresh tumor specimens without single-cell dissociation (Jacob et al. 2020). With this new protocol, they set up a biobank of 70 GBOs from 53 patients, with a success rate of 66.7%-91.4%, which is better than establishing glioblastoma stem cell lines. They performed systematic histological, transcriptional, and mutational profiling of these tumor organoids to demonstrate their faithfulness in recapitulating the original tumors, and showed that these GBOs could be widely used for xenografts and targeted drug testing. Remarkably, co-culturing GBOs with CAR-T cells can also model immunotherapy responses.

#### 3D Multicellular coculture models for GBM

Building upon their organoid cell-of-origin models for GBM, Ogawa et al. co-cultured organoid-derived tumor cells or primary patient-derived glioblastoma cell lines with mature organoids to show that they can establish invasive tumor-like structures (Ogawa et al. 2018). Along this line, Linkous et al. and Azzarelli et al. established 3D coculture of GSCs with pluripotent stem cell-derived brain organoids (Azzarelli et al. 2021; Linkous et al. 2019). Both studies found that GSCs can integrate into the host organoid, Linkous et al. further demonstrated that they could form microtubular invasive “glioma networks” (Linkous et al. 2019). Thus, these models can be used for rapid and efficient GBM invasion assays, as described in several studies (Choe et al. 2020; Goranci-Buzhala et al. 2020; Krieger et al. 2020). Moreover, these models offer a platform to efficiently test the tumorigenicity of subpopulations of glioma cells in a human host microenvironment, as demonstrated in a recent study that identified PTPRZ1^+^ outer radial glial-like cancer stem cells in GBM (Bhaduri et al. 2020).

In addition to these “tumor-in-the-organoid” coculture models, 3D multicellular coculture systems using different cellular compositions have been explored to mimic the native cellular and immune microenvironment. Plummer et al. established a 3D heterotypic GBM brain sphere model by coculturing glioblastoma tumor cells, IPSC-derived neurons, glial cells and astrocytes into a spheroid (Plummer et al. 2019). Tang et al. utilized 3D bioprinting to generate GBM tissue models with GSCs, astrocytes, neural stem/progenitor cells, and IPSC-derived macrophage (Tang et al. 2020). While not exactly a pluripotent stem cell-derived model, Cornelison et al. constructed a four-component 3D model coculturing GSCs, human primary human astrocytes, and immortalized human microglia (Cornelison et al. 2022). These models are useful alternatives to GBM-organoid coculture models, particularly in that they include important cell types that are commonly missing in brain organoids.

### Diffuse Midline Glioma, H3K27M-mutant (DMG) and H3G34R-mutant High-grade Glioma (HGG)

DMG is a new entity added to malignant gliomas in the latest WHO classification (Louis et al. 2021), which includes the tumor type formerly known as Diffuse Intrinsic Pontine Glioma (DIPG). This lethal tumor most commonly occurs in children, typically in the hindbrain midline regions such as the brain stem, cerebellum, and spinal cord. It harbors a signature lysine 27-to-methionine mutation in histone H3 (H3K27M), often accompanied by *TP53* mutations and *PDGFRA* amplification (Wu et al. 2012). In contrast, glycine 34-to-arginine mutation in histone H3 (H3G34R) is associated with forebrain-derived, non-midline hemispheric HGG that most commonly occurs in children and young adults (Mackay et al. 2017). Compared to GBM, these tumors lack tissue samples and cell lines partly due to lower disease occurrence and their sensitive anatomic locations in important brain regions.

Recently, a series of cell-of-origin models using human stem cells have been developed to provide novel insights into these understudied malignant gliomas. In a pioneering work, Funato et al. showed that *TP53* loss coupled with overexpression of *H3K27M* and *PDGFRA* were sufficient to drive the malignant transformation of human ESC-derived NSCs after orthotopic transplantation (Funato et al. 2014). The notion that embryonic NSCs, but not OPCs, are the cell-of-origin for DMG was further confirmed by subsequent studies using cell-of-origin models and GEM models for DMG (Haag et al. 2021; Larson et al. 2019; Pathania et al. 2017).

H3K27M-mutant DMG and H3G34R-mutant HGG are both driven by oncohistones but arise in discrete brain regions (forebrain and hindbrain, respectively). To investigate the underlying mechanism, Bressan et al. and Funato et al. independently compared the oncogenic effects of H3K27M and H3G34R on both forebrain and hindbrain fetal human NSCs using similar cell-of-origin models (Bressan et al. 2021; Funato et al. 2021). Both studies demonstrated that H3K27M and H3G34R preferentially increase the tumorigenicity of hindbrain and forebrain NSCs, respectively, and identified downstream events responsible for these regional differences.

### Low-grade Glioma (LGG)

LGG is classified as a WHO grade II tumor, which includes low-grade astrocytoma, oligodendroglioma, and mixed glioma (Louis et al. 2021). LGG is the slowliest-growing glioma in adults, making it much more difficult to establish cell lines and PDX models compared to HGG. Modrek et al. established cell-of-origin models for LGG by introducing common LGG driver mutations *IDH1*/*TP53*/*ATRX* into human NSCs (Modrek et al. 2017), although it remains debatable whether human LGGs are derived from NSCs or more mature cells such as astrocytes. Abdullah et al. adapted the tumor organoid protocol for HGG to culture LGG primary tissue samples by utilizing physiologic (5%) oxygenation conditions (Abdullah et al. 2022). With this protocol, they achieved a remarkable success rate of 87% (13/15) to establish the first LGG organoid models that mimic parental tumors at cellular and genetic levels, paving the way for further preclinical studies.

### Neurofibromatosis Type 1 (NF1) and Neurofibromatosis Type 2 (NF2)

NF1 is a familiar cancer-predisposing syndrome caused by germline mutations in the *NF1* gene (Gutmann et al. 2017; Louis et al. 2021). Patients with NF1 are prone to develop peripheral nerve sheath tumors called neurofibromas, a type of glioma in the peripheral nervous system (PNS). GEM studies have shown that neurofibromas most likely originate from Schwann cell lineage cells (Chen et al. 2014; Chen et al. 2019; Zhu et al. 2002), which are a PNS counterpart of OPC/oligodendrocyte in the CNS. Since NF1 is a germline-inherited disease, it makes sense to develop cell-of-origin models for neurofibroma using NF1 patient-derived IPSCs. Two studies have shown that *NF1*-/- Schwann cell lineage cells differentiated from patient IPSCs can develop into neurofibromas in immunocompromised mice (Mazuelas et al. 2022; Mo et al. 2021).

Similar to NF1, NF2 is a genetic condition caused by mutations in the *NF2* gene (Coy et al. 2020). Patients with NF2 frequently develop Schwannomas on the vestibular nerve (Vestibular Schwannoma, VS) in the inner ear, meningiomas, and ependymomas (Coy et al. 2020). Although there is currently a lack of NF2 stem cell/organoid models, inner ear organoids with full sensory circuits and myelinating Schwann cells have been reported in a preprint study (Steinhart 2021; Valk et al. 2021), paving the way for the development of cell-of-origin models for NF2-associated VS.

### Medulloblastoma (MB)

MB is the most common brain tumor in children that usually originate in the cerebellum (Louis et al. 2021). MBs are divided into four molecular subgroups: WNT, SHH, Group 3, and Group 4, each exhibit a distinct mutational/transcriptional spectrum and has unique cell(s)-of-origin demonstrated by GEM models (Northcott et al. 2012; Taylor et al. 2012). The WNT subgroup has the best prognosis (> 95%), which harbors somatic mutations of *CTNNB1* and likely originates from neural progenitor cells of the lower rhombic lip (Gibson et al. 2010). The SHH subgroup (overall survival of 60%-80%) is characterized by mutations of *PTCH1* and *SUFU*, negative regulators of the SHH pathway (Taylor et al. 2012). These tumors most likely originate from cerebellar granule neuron precursors (CGNPs) of the external granular layer, or NSCs in the SVZ of the 4^th^ ventricle (Schuller et al. 2008; Yang et al. 2008). Group 3 has the worst prognosis (~ 50%), is characterized by high-level *MYC* amplification and may arise from CGNPs or Prominin1-positive, lineage-negative NSCs (Kawauchi et al. 2012), (Pei et al. 2012). Group 4 is the most common (~ 40%) MB subgroup, driven by *MYCN* and *CDK6*, yet there is no faithful preclinical models for this subgroup.

Cell-of-origin models for MB built upon the finding in GEM models to introduce MB drivers into human NSCs to initiate MB development. In 2016, Hanaford et al. develop the first human NSC-derived model for Group 3 MB. They transduced human NSCs from the cerebellar anlage with overexpression of c-MYC, dominant-negative p53, constitutively active AKT and hTERT (Hanaford et al. 2016). This genetically modified human NSC line can develop into Group 3 MB-like tumors after xenograft. However, one might argue that this genetic combination may not accurately reflect the genomics of Group 3 MB. In 2021 Xue et al. established a genetically more relevant cell-of-origin model for Group 3 MB by transforming human IPSC-derived NSCs with *MYC* overexpression and *TP53* loss (Xue et al. 2021). For the SHH MB subgroup, cell-of-origin models were developed by introducing *MYCN* overexpression in human neuroepithelial stem cells (Čančer et al. 2019; Huang et al. 2019), or using IPSCs from Gorlin syndrome patients carrying germline *PTCH1* mutations (Huang et al. 2019; Susanto et al. 2020). Notably, Huang et al. found that their MYCN-driven tumors were more representative of human MB compared to an MYCN-driven GEM model in terms of transcriptomes and DNA methylation patterns (Huang et al. 2019). All of these models have been useful to identify downstream/cooperative molecular targets and evaluate drug responses.

Currently, there are only a few studies using organoids to model MB. Ballabio et al. established the first human cerebellar organoid-derived cell-of-origin models for MB (Ballabio et al. 2020). They tested several gene combinations in GEM models and cerebellar organoids, and identified OTX2 and c-MYC as strong inducers of Group3 MB-like tumors, which are sensitive to an EZH2-specific inhibitor Tazemetostat. For MB tumor organoids, there is currently only one published report using MB cell lines to generate tumor organoids (Frisira et al. 2019). A recent preprint in bioArxiv may be the first study to establish MB organoids using resected tumor specimens (Yuchen Li 2022), following an adapted protocol of culturing GBM tumor organoids (Jacob et al. 2020).

### Atypical Teratoid/rhabdoid Tumor (AT/RT)

AT/RT is a lethal childhood malignant CNS tumor (Louis et al. 2021). It harbors signature *SMARCB1* mutations and exhibits rhabdoid cell histology. Studies in GEM models demonstrated that AT/RT may develop from *Smarcb1*-mutant embryonic neural stem/progenitor cells (Han et al. 2016; Ng et al. 2015). Based on this knowledge, Terada et al. established cell-of-origin models for AT/RT by knockout of *TP53* and *SMARCB1* in ESCs, and uncovered the ESC transcriptional signature as a key driver for AT/RT (Terada et al. 2019). There are currently no reported AT/RT tumor organoid models.

### Meningioma

Meningioma is the most common primary CNS tumor that arises from arachnoid cap cells in the meninges (Louis et al. 2021). 80% of them are benign WHO Grade I tumors, yet about 10% are atypical (grade II) or anaplastic (grade III) forms. Meningiomas are generally slow-growing tumors on the surface of the brain or spinal cord. These tumors may compress adjacent brain tissue as they grow, requiring surgical removal. 40% to 60% of sporadic meningiomas are *NF2*-inactivated (Riemenschneider et al. 2006). Comprehensive genomic profiling revealed additional tumor driver mutations in *TRAF7*, *KLF4*, *AKT1*, and *SMO* (Clark et al. 2013). Similar to LGG, cell lines and PDX models for meningioma are few. In 2021, Yamazaki et al. reported the first tumor organoid models for meningioma with a 100% success rate (two malignant, twelve benign) (Yamazaki et al. 2021). As proof of principle, they used these models to dissect the function of *FOXM1,* a meningioma-relevant gene, in the maintenance of these tumor organoids. There are currently no cell-of-origin models for meningioma, possibly due to the technical difficulties in culturing the meninges as cell lines or organoids.

## Opportunities and challenges for precision nervous system tumor modeling

Human stem cell/organoid-derived models have provided new tools for precision nervous system tumor modeling. Compared to GEM models, they are more human-relevant since they directly model the behavior of human cells, and do not require time-consuming multi-generation crossing. Compared to 2D cancer cell lines and PDX models, cell-of-origin models offer unique insights into the de novo tumorigenesis from genetically defined tumor-initiating human cells, tumor organoid models better preserve the genetic/transcriptional diversity of parental tumors and generally have a higher success rate to establish, while 3D multicellular coculture models reconstitute the complex human tumor microenvironment in a 3D setting. These models are especially valuable for the study of tumor types wherein few GEM and PDX models are available.

While human stem cell/organoid-derived models bring new opportunities for understanding the tumorigenesis and progression of nervous system tumors, there are several challenges that need to be addressed in the next phase development of these models.

For human stem cell-based models, one potential problem is that similar to PDX models, they require xenograft into immunocompromised mice, and essentially developing tumors in a mouse brain microenvironment. To make these models more human-relevant, we could use humanized mice through engraftment of human cell types such as microglia and other immune cells.

For human brain organoid-based models, a key issue is that they miss key cell types in the tumor microenvironment. Unlike epithelial organoids that can be derived from both adult stem cells and pluripotent stem cells, currently brain organoids can only be generated from pluripotent stem cells (Clevers 2016; Tang et al. 2022). Consequently, brain organoids more closely resemble immature fetal brains lacking mature neurons and glial cells, putting into question how closely they mimic the tumor microenvironment of adult onset brain tumors such as GBM. Other important missing cell types in the brain organoids are non-neural cells such as endothelial cells and immune cells (Cakir and Park 2022). Efforts have been made to introduce mature cell types such as myelinating oligodendrocytes in brain organoids (Madhavan et al. 2018), and there are already reports on vascularized and immunized brain organoids (Abud et al. 2017; Cakir et al. 2019; Sun et al. 2022). However, these types of more complex brain organoids are yet to be used to model nervous system tumors.

While human stem cell/organoid-derived models are in theory very promising for drug screening, it is yet to be demonstrated that these screening schemes are more suitable or more predictive of patient responses than less expensive cell-based multi-well-plate screenings in a head-to-head comparison. As a step in this direction, Jabs et al. showed that drug effects in organoids were more diverse than in cell lines (Jabs et al. 2017). More detailed comparative studies are needed for establishing human stem cell/organoid-derived models as a reliable and efficient drug screening platform.

## Conclusions

Human stem cell/organoid-derived models start a new era of precision nervous system tumor modeling, and the field is rapidly evolving. Looking forward, we would expect that more models will be developed for different types of nervous system tumors using different genetic combinations and initiating cell types. The current organoid models could be further optimized by integrating more diverse cell types as tumor-initiating cells or tumor microenvironmental cells to study the complex process of tumorigenesis. Together, these technological advancements will hopefully help establish more faithful models for the development of life-saving therapies.

## Data Availability

Not applicable.

## Abbreviations

AT/RT: Atypical teratoid/rhabdoid Tumor
CGNP: Cerebellar granule neuron precursor
DIPG: Diffuse Intrinsic Pontine Glioma
DMG: Diffuse Midline Glioma
ESC: Embryonic stem cell
GBO: GBM organoid
GEM: Genetically engineered mouse model
GBM: Glioblastoma
GSC: Glioma stem cell
H3G34R: Glycine 34-to-arginine mutation in histone H3
HGG: High-grade Glioma
IPSC: Induced pluripotent stem cell
LGG: Low-grade Glioma
H3K27M: Lysine 27-to-methionine mutation in histone H3
MB: Medulloblastoma
MYC^OE^: MYC overexpression
NES: Neuroepithelial stem cells
NSC/NPC: Neural stem/progenitor cell
NF1: Neurofibromatosis Type 1
NF2: Neurofibromatosis Type 2
OPC: Oligodendrocyte precursor cells
PDX: Patient-derived xenograft
PNS: Peripheral nervous system
CNS-PNET: Primitive neuroectodermal tumors of the central nervous system
SVZ: Subventricular zone
3D: Three-dimensional
VS: Vestibular Schwannoma
